# Association between gout and subsequent breast cancer: a retrospective cohort study including 67,598 primary care patients in Germany

**DOI:** 10.1007/s10549-023-06944-w

**Published:** 2023-04-18

**Authors:** Niklas Gremke, Sebastian Griewing, Karel Kostev, Uwe Wagner, Matthias Kalder

**Affiliations:** 1 Department of Gynecology and Obstetrics, University Hospital Marburg, Philipps-University Marburg, Baldingerstraße, 35043 Marburg, Germany; 2 IQVIA, Main Airport Center, Unterschweinstiege 2–14, 60549 Frankfurt, Germany

**Keywords:** Breast cancer, Epidemiology, Risk factors, Primary care, Germany, Inflammation, Hyperuricemia, Gout

## Abstract

**Purpose:**

The aim of this retrospective cohort study was to analyze the cumulative incidence of breast cancer following gout and to investigate the association between gout and subsequent breast cancer in 67,598 primary care patients in Germany.

**Methods:**

This study included adult female patients (≥ 18 years) with an initial diagnosis of gout in 1284 general practices in Germany between January 2005 and December 2020. Individuals without gout were matched to gout patients using propensity score matching based on average yearly consultation frequency during the follow-up period, diabetes, obesity, chronic bronchitis/COPD diagnoses, and diuretic therapy. The 10-year cumulative incidence of breast cancer in the cohorts with and without gout was also studied using Kaplan–Meier curves, which were then compared using the log-rank test. Finally, a univariable Cox regression analysis was conducted to assess the association between gout and breast cancer.

**Results:**

After up to 10 years of follow-up, 4.5% of gout and 3.7% of non-gout patients were diagnosed with breast cancer. A Cox regression analysis revealed a significant association between gout and subsequent breast cancer in the total population (HR: 1.17; 95% CI: 1.05–1.31). In the age-stratified analyses, gout was only strongly associated with subsequent breast cancer in the age group ≤ 50 (HR: 1.58; 95% CI: 1.10–2.27), but the association was not significant in women over 50 years old.

**Conclusion:**

Taken together, the findings of our study provide evidence for the association between gout and subsequent breast cancer diagnosis, particularly in the youngest age group.

## Introduction

Breast Cancer (BC) is the most common cancer type in women worldwide, with an estimated 2.1 million newly diagnosed cases in 2018 [1]. In Germany, approximately 68,950 women were diagnosed with BC in 2016 and 18,570 women died of the disease [2]. The number of BC cases worldwide is expected to increase to 2.7 million annually by 2030 [3–5]. Notably it has been estimated that about 20% of all BC-related deaths can be attributed to modifiable risk factors such as alcohol use, obesity (BMI ≥ 30), and reduced physical activity [6]. For example, obesity is not only linked with increased BC incidence, but is also associated with a poor outcome in terms of increased BC recurrence and reduced survival compared to normal-weight BC patients [7–9]. Recent studies support the hypothesis that obesity-related inflammation is a major contributor to this association [10]. In particular, it has been well documented that systemic inflammation as a hallmark of cancer plays a crucial role in breast carcinogenesis and progression [11]. It is also hypothesized that physical activity lowers BC risk in postmenopausal women by lowering systemic inflammatory markers, including TNF-α, IL-6, and C-reactive protein (CRP) [12, 13].

In view of the above, the association between BC and gout as a common inflammatory disease has attracted much interest during the past few years. Briefly, gout is the most common form of inflammatory arthritis worldwide. Despite being one of just a few curable rheumatic diseases, both the prevalence and incidence of gout are increasing across the globe so that it is being hypothesized in the literature that a modern gout epidemic is currently developing that is on a similar scale to the obesity epidemic [14, 15]. This increasing disease burden of gout is a consequence of lifestyle changes, obesity and dietary factors. However, genetic polymorphisms are also responsible for the development of gout and hyperuricemia [16–18]. Mechanistically, the pathogenesis of gout can be divided into different phases: The initial development of hyperuricemia (serum urate > 6.8 mg/dl), followed by the deposition of monosodium urate crystals (MSU) usually in tissues with low temperature or acidic pH, such as joints and periarticular structures [19]. Finally, MSU crystal deposition can lead to an acute gout flare, particularly at the first metatarsophalangeal joint, the midfoot, and the knee with the rapid onset of painful acute inflammatory arthritis characterized by a swollen, hot, and red joint [20, 21]. Notably, a gout flare is a clinical diagnosis, but the diagnosis is also generally considered to be accurate when made in a primary care setting using well-validated diagnostic criteria [22].

In the past, *Strasak *et al*.* showed in a prospective study including more than 28,000 older Austrian women (aged ≥ 50 years) that hyperuricemia (> 5.41 mg/dL) was independently associated with an increased risk of total cancer mortality (HR: 1.27, 95% CI 1.08–1.48). In particular, malignant neoplasms of the breast and female genital organs were positively associated with hyperuricemia [23]. In line with these results, a metanalysis of three different prospective cohort studies with a total of 50,358 patients revealed that gout patients were at an increased risk of cancer, particularly urological cancers, digestive system cancers, and lung cancer. However, due to several study limitations of these works (e.g., limited sample size), the association between gout and breast cancer remains unclear [24–27].

Aiming to explore this topic in more detail, we conducted a retrospective cohort study including 67,598 primary care patients in Germany to investigate the cumulative incidence of breast cancer as a function of gout and to explore the association between gout and subsequent breast cancer using univariable Cox regression analysis.

## Methods

### Database

This retrospective cohort study was based on data from the Disease Analyzer database (IQVIA), which contains drug prescriptions, diagnoses, and basic medical and demographic data obtained directly and in anonymous format from computer systems used in the practices of general practitioners and specialists [28]. The database covers approximately 3% of all private practices in Germany. The sampling method for the Disease Analyzer database is based on summary statistics from all doctors in Germany published yearly by the German Medical Association. IQVIA uses these statistics to determine the panel design based on four strata including specialist group, German federal state, community size category, and age of physician. It has previously been shown that the panel of practices included in the Disease Analyzer database is representative of general and specialized practices in Germany. Finally, this database has already been used in previous studies focusing on breast cancer [29–31].

### Study population

**Fig. 1:**
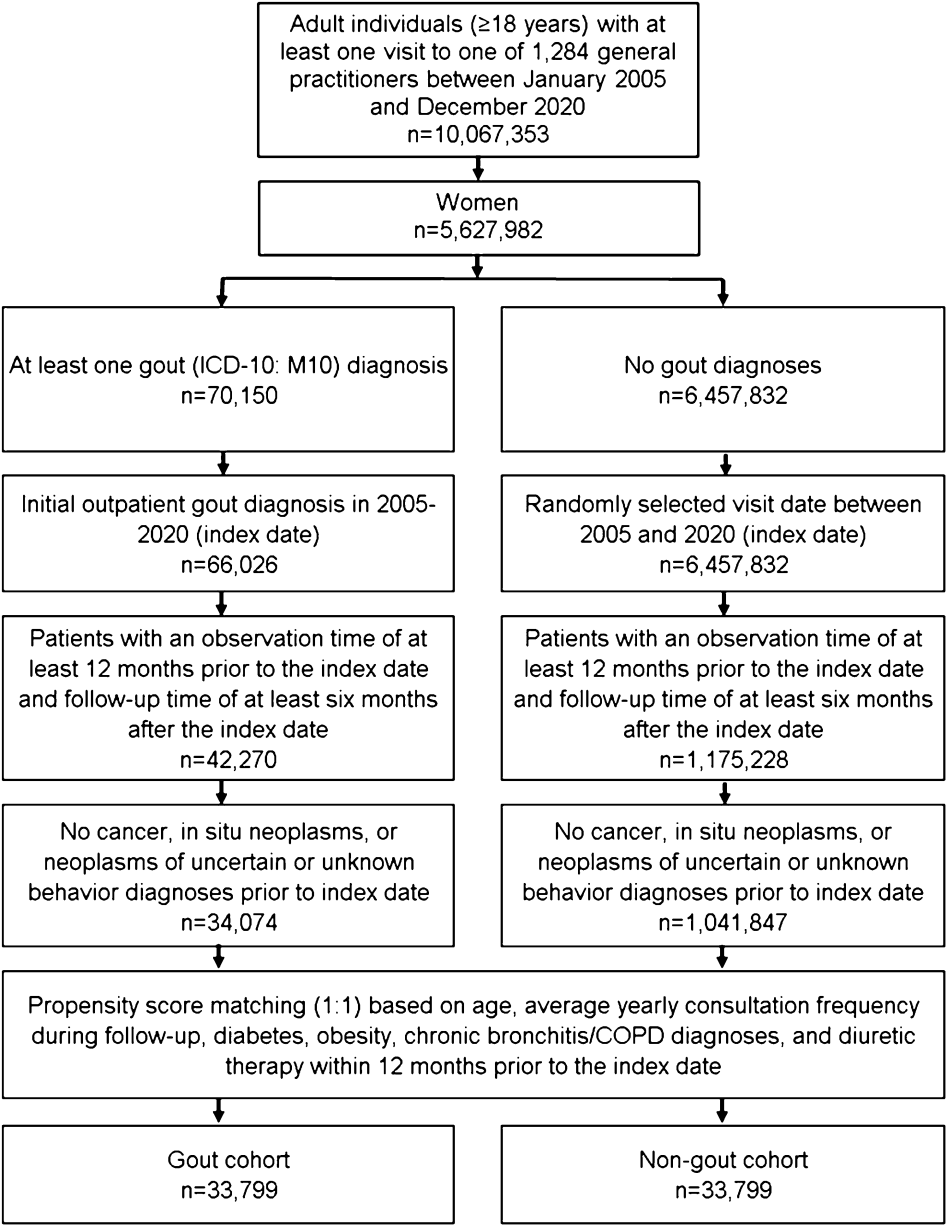
Selection of study patients

This study included adult female patients (≥ 18 years) with an initial diagnosis of gout (ICD-10: M10) in 1,284 general practices in Germany between January 2005 and December 2020 (index date; Fig. 1). Further inclusion criteria included an observation time of at least 12 months prior to the index date and a follow-up time of at least 6 months after the index date. Patients with diagnoses of cancer (ICD-10: C00–C97), in situ neoplasms (ICD-10: D00–D09), and neoplasms of uncertain or unknown behavior (ICD-10: D37–D48) prior to or on the index date were excluded.

After applying similar inclusion criteria, individuals without gout were matched to gout patients using propensity score matching (1:1) based on average yearly consultation frequency during the follow-up period, diabetes, obesity, chronic bronchitis/COPD diagnoses, and diuretic therapy within 12 months prior to the index date. For the non-gout cohort, the index date was that of a randomly selected visit between January 2005 and December 2020 (Fig. 1). Diabetes (ICD-10: E10–E14) and obesity (ICD-10: E66) were included in matching as these diagnoses are associated with breast cancer. As the database used does not contain information on smoking status, chronic bronchitis (ICD-10: J42) and COPD (ICD-10: J44) diagnoses were used in matching due to their strong association with smoking behavior [32]. Finally, diuretic therapy was also included in matching as it is a known risk factor for gout and the prevalence of diuretic prescription was much higher among gout patients [33].

### Study outcome and statistical analyses

The outcome of the study was the initial diagnosis of breast cancer (ICD-10: C50) in the period of up to 10 years following the index date as a function of gout. Differences in the sample characteristics and diagnosis prevalence between the gout and non-gout cohorts were compared using the Wilcoxon signed-rank test for continuous variables, the McNemar test for categorical variables with two categories, and the Stuart-Maxwell test for categorical variables with more than two categories.

The 10-year cumulative incidence of breast cancer in the cohorts with and without gout was also studied using Kaplan–Meier curves which were then compared using the log-rank test. Finally, a univariable Cox regression analysis was conducted to assess the association between gout and breast cancer. The results of the Cox regression model are displayed as hazard ratios (HRs) and 95% confidence intervals (CIs). In addition, Cox regression analyses were conducted separately for different age groups. *P* values of < 0.05 were considered statistically significant. Analyses were carried out using SAS version 9.4 (SAS Institute, Cary, USA).

## Results

### Basic characteristics of the study sample

The present study included 33,799 women with gout and 33,799 women without gout. The basic characteristics of study patients are displayed in Table 1. The mean age was 66 years. Patients visited their GPs an average of 6.9 times per year during the follow-up period. A high proportion of patients (45.2%) received diuretic prescriptions within 12 months prior to the index date. Of 33,799 gout patients, 71% had a diagnosis of unspecified gout (ICD-10: M10.9), and 27% were diagnosed with idiopathic gout (ICD-10: M10.0). Other gout types were extremely rare.

**Table 1:** Baseline characteristics of the study sample (after propensity score matching)

Variable	Proportion among gout patients (%) *N* = 33,799	Proportion among non-gout patients (%) *N* = 33,799	*P*-value
Age (Mean, SD)	65.6 (15.7)	59.4 (14.5)	0.979
Age ≤ 50	17.2	17.2	0.999
Age 51**–**60	17.5	17.5	
Age 61**–**70	20.5	20.5	
Age > 70	44.8	44.8	
Number of physician visits per year during the follow-up (Mean, SD)	6.9 (4.6)	6.9 (4.6)	0.946
Diabetes	39.4	39.4	1.000
Obesity	19.9	19.9	0.832
Chronic bronchitis/COPD	15.9	16.0	0.817
Diuretic prescriptions	45.2	45.2	0.865

Proportions of patients given in % unless otherwise indicated. SD: standard deviation

### Association between gout and subsequent breast cancer diagnosis

**Fig. 2:**
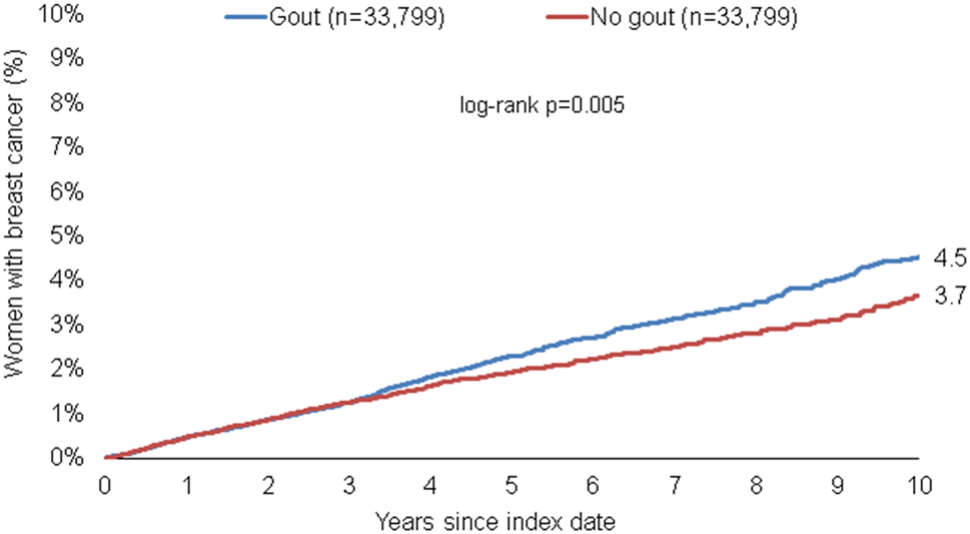
Cumulative incidence of breast cancer in patients with and without gout

After up to 10 years of follow-up, 4.5% of the gout and 3.7% of the non-gout cohort were diagnosed with breast cancer (Fig. 2). This resulted in an incidence of 4.5 versus 3.9 cases per 1000 patient-years.

The regression analysis showed a significant association between gout and subsequent breast cancer in the overall population (HR: 1.17; 95% CI: 1.05–1.31). In the age-stratified analyses, gout was only strongly associated with a subsequent breast cancer diagnosis in the age group ≤ 50 (HR: 1.58; 95% CI: 1.10–2.27), but the association was not significant in women over 50 years old (Table 2).

**Table 2:** Association between gout and subsequent breast cancer diagnosis in patients followed in general practices in Germany (univariable Cox regression models)

Outcome diagnosis	Incidence (cases per 1000 patient-years) among gout cohort	Incidence (cases per 1000 patient-years) among non-gout cohort	HR for gout (95% CI)	*P*-value
Total	4.5	3.9	**1.17 (1.05–1.31)**	**0.005**
Age ≤ 50	2.7	1.7	**1.58 (1.10–2.27)**	**0.014**
Age 51**–**60	4.2	3.9	1.08 (0.83–1.39)	0.582
Age 61**–**70	4.7	4.0	1.21 (0.96–1.52)	0.112
Age > 70	5.5	4.9	1.14 (0.97–1.33)	0.116

Bold HR’s and *p*-values are statistically significant (*p* < 0.05)

## Discussion

Our study revealed differences in the cumulative breast cancer incidence between the gout and non-gout cohorts. The Cox regression analysis indicated a significant association between gout and subsequent breast cancer in the overall population. In line with our results, Strasak and colleagues reported in a prospective study that high serum uric acid (SUA) levels (> 5.41 mg/dL) were independently associated with an increased risk of total cancer mortality (*p* < 0.0001). In particular, the authors found an independent, positive association with deaths due to malignant neoplasms of the breast and female genital organs [23]. In addition, Levine and colleagues reported a positive association between SUA levels and total cancer mortality among females aged 55–64 years, even after adjustment for multiple risk factors (e.g., age, relative weight, smoking) [34]. Despite being the main characteristic of gout, hyperuricemia is only one factor of many that contribute to the transition from hyperuricemia to clinically evident gout [16]. However, it has been shown that patients with established gout also have an increased risk of cancer [25–27, 35]. In particular, a national population study from Taiwan showed that the annual incidence of cancer in gout patients was more than twice as high as in the normal population (8.7 vs. 4.2 cases per 1000 patient-years, *p* < 0.001). Gout was most closely associated with prostate cancer, with an age- and sex-adjusted HR of 1.71 (1.45–2.02) [25]. Another retrospective cohort study from Taiwan revealed that gout patients are more likely to develop urological cancers such as prostate, bladder, and renal cancers in particular [26]. Finally, Boffetta and colleagues showed in a large prospective study of 16,857 gout patients admitted to hospitals in Sweden that the incidence of cancers of the oral cavity and pharynx, colon, liver and biliary tract, pancreas, lung, skin (melanoma and nonmelanoma), endometrium, and kidney was higher among gout patients [27]. In summary, gout is associated with a broad spectrum of different tumor entities, meaning that the prevalent type of cancer in gout patients could be dependent on culture, vary between countries, and may also be influenced by tumor site-specific risk factors [36].

In the past, the literature has indicated that SUA exhibits antioxidant properties by scavenging reactive oxygen species (ROS) and, therefore, protects against cancer [37]. However, today there is increasing evidence that SUA plays a crucial role as a pro-oxidant by promoting inflammatory reactions and oxidative stress, thereby contributing to cancer development. Focusing on the pro-tumorigenic role of SUA in breast cancer progression, it is hypothesized that SUA might be partially responsible for the low-grade inflammation in the breast tumor microenvironment that contributes to tumor cell proliferation and metastasis [38]. Contrary to this, a prospective population-based study revealed that SUA levels were significantly inversely associated with breast cancer risk and overall cancer mortality, but not with the risk of lung, prostate, and colorectal cancer. However, if the antioxidant potential of uric acid is an underlying cause of the observed associations between SUA and breast cancer and cancer mortality, it would be reasonable to expect that such a universal mechanism might also cause similar associations with other cancer types and not just with breast cancer [39].

Finally, the age-stratified analyses in our study revealed that gout was only strongly associated with subsequent breast cancer in the age group ≤ 50 (HR: 1.58; 95% CI: 1.10–2.27). Interestingly, a national population-based study from Korea found a higher risk of cancer, as well as both all-cause and cancer mortality, in middle-aged patients (41–55 years) with gout compared to the general population. The subgroup analysis of this study showed that the risk of stomach cancer, head, and neck cancer and hematologic or lymphoid cancers was higher in middle-aged gout patients than in controls, whereby the risk of BC was not calculated. Notably, these results are in line with our observation that middle-aged gout patients have the highest cancer risk compared to controls [36].

Based on the fact that the median age at menopause among women from industrialized countries ranges between 50 and 52 years [40], one can assume that the menopause status may explains the exclusive significant association of gout and BC in women aged < 50. Indeed, it is known that postmenopausal women experience a higher occurrence of gout, while estrogen's uricosuric effect shields premenopausal women [41]. Given the positive association between hyperuricemia and higher cancer incidence (incl. BC), these results suggest that BC should be more prevalent in gout patients aged > 50 [24]. However, we observed the opposed effect. Notably, conflicting results have been reported in other studies focusing on gout incidence and menopausal status. According to an analysis of National Health and Nutrition Examination Survey data from 1999 to 2010, the occurrence of hyperuricemia was not associated with menopause [42]. Finally, further research is required to examine how hyperuricemia and gout are linked to breast cancer, considering factors such as age, inflammation, and menopausal status.

## Strengths and limitations

Our retrospective cohort study has several strengths: The german disease analyzer (DA) is a large European outpatient database containing data from 2898 practices with about 7.8 million patients in Germany. The representativeness of the diagnoses it contains has already been validated [43, 44]. Furthermore, DA provides continuously updated data generated directly from practice computers based on patient data (diagnoses, demographic data, prescriptions, etc.) and has successfully been used for several studies in various disciplines [43, 45–47]. Notably, we performed propensity score matching based on diabetes and obesity as it is well known that both are strongly related to breast cancer [48]. We also included the use of diuretics in the matching process based on their tendency to induce hyperuricemia which can in turn lead to the development of gout. Furthermore, patients diagnosed with cancer prior to the index date were excluded since hyperuricemia (due to increased urate overproduction) can occur in patients with disorders involving high cell turnover such as advanced tumor diseases or highly proliferative myeloproliferative disorders [49, 50]. However, the DA does not contain any information on external confounding factors (e.g., alcohol and socioeconomic status). Importantly, alcohol consumption and lifestyle factors are important risk factors for developing gout and no information on them was available for this study [15]. Also, SUA levels are not recorded in the DA database and the association between hyperuricemia and BC risk, therefore, could not be evaluated. Finally, there is also a lack of hospital data and information on mortality.

## Conclusion

In conclusion, our study provides evidence for the association between gout and subsequent breast cancer diagnosis, particularly in the youngest age group.

## Data Availability

Anonymized raw data are available upon reasonable request.
